# Supplementary material for: Clinical evaluation of platelet-rich plasma therapy for osteonecrosis of the femoral head: A systematic review and meta-analysis
Source: PLoS One. 2024 May 24;19(5):e0304096. doi: 10.1371/journal.pone.0304096 (PMC11125492; doi:10.1371/journal.pone.0304096)
Supplement: S1 Table — (PDF) [file pone.0304096.s001.pdf]

Supplementary table1. Search strategy.

Database	Step	Search term
PubMed	#1	((platelet-rich plasma [Title/Abstract]) OR (platelet-rich [Title/Abstract]) OR (platelet rich plasma [Title/Abstract])
	#2	("osteonecrosis of the femoral head" [Mesh]) OR ((femur head necrosis [Title/Abstract])) OR (ONFH [Title/Abstract])) OR (FHN [Title/Abstract])
	#3	#1 AND #2
Web of Science	#1	TS=(platelet-rich plasma OR platelet-rich OR platelet rich plasma)
	#2	TS=(osteonecrosis of the femoral head OR femur head necrosis OR ONFH OR FHN)
	#3	#1 AND #2
Embase	#1	'platelet-rich plasma'/exp
	#2	'platelet-rich':ab,ti OR 'platelet rich plasma':ab,ti
	#3	#1 OR #2
	#4	'osteonecrosis of the femoral head'/exp
	#5	'femur head necrosis':ab,ti OR 'ONFH':ab,ti OR 'FHN':ab,ti
	#6	#4 OR #5
	#7	#3 AND #6
Cochrane Central Register of Controlled Trials	#1	(platelet-rich plasma):ti,ab,kw OR (platelet-rich):ti,ab,kw OR (platelet rich plasma):ti,ab,kw
	#2	MeSH descriptor: [osteonecrosis of the femoral head] explode all trees
	#3	(femur head necrosis):ti,ab,kw OR (ONFH):ti,ab,kw OR (FHN):ti,ab,kw
	#4	#2 OR #3
	#5	#1 AND #4
Chinese National Knowledge Infrastructure	#1	(((主题=富血小板血浆 或者 题名=富血小板血浆 或者 v_subject=中英文扩展(富血小板血浆) 或者 title=中英文扩展(富血小板血浆)) 或者 (主题=富血小板 或者 题名=富血小板 或者 v_subject=中英文扩展(富血小板) 或者 title=中英文扩展(富血小板))) 或者 ((主题=PRP 或者 题名=PRP 或者 v_subject=中英文扩展(PRP) 或者 title=中英文扩展(PRP)))
	#2	((主题=股骨头坏死 或者 题名=股骨头坏死 或者 v_subject=中英文扩展(股骨头坏死) 或者 title=中英文扩展(股骨头坏死)) 或者 (主题=股骨头缺血性坏死 或者 题名=股骨头缺血性坏死 或者 v_subject=中英文扩展(股骨头缺血性坏死) 或者 title=中英文扩展(股骨头缺血性坏死)))
	#3	#1 AND #2
Chinese Science and Technology Periodical database	#1	(题名或关键词=富血小板血浆 OR 题名或关键词=富血小板 OR 题名或关键词=PRP)
	#2	题名或关键词=股骨头坏死 OR 题名或关键词=股骨头缺血性坏死
	#3	#1 AND #2
WanFang database	#1	(主题:(富血小板血浆) or 主题:(富血小板) or 主题:(PRP)
	#2	(主题:(股骨头坏死) or 主题:(股骨头缺血性坏死)
	#3	#1 AND #2
Chinese Biological Medicine database	#1	"富血小板血浆"[不加权:扩展]
	#2	"富血小板"[常用字段:智能] OR "PRP"[常用字段:智能]
	#3	#1 OR #2
	#4	"股骨头坏死"[常用字段:智能] OR "股骨头缺血性坏死"[常用字段:智能]
	#5	#3 AND #4
