# Supplementary material for: Clinical evaluation of platelet-rich plasma therapy for osteonecrosis of the femoral head: A systematic review and meta-analysis
Source: PLoS One. 2024 May 24;19(5):e0304096. doi: 10.1371/journal.pone.0304096 (PMC11125492; doi:10.1371/journal.pone.0304096)
Supplement: S3 Table — (PDF) [file pone.0304096.s003.pdf]

Supplementary table 3. Seneitivity analysis for Harris hip score.

Eliminated study	Heterogeneity		Effect Model	MD	95% CI	P Value
	P Value	I^2 (%)				
None	<0.00001	88	Random	6.57	4.81 to 8.33	<0.00001
Aggarwal 2020 [31]	<0.00001	89	Random	6.22	4.47 to 7.97	<0.00001
Chai 2022 [27]	<0.00001	89	Random	6.72	4.82 to 8.62	<0.00001
Dai 2019 [35]	<0.00001	88	Random	6.30	4.50 to 8.10	<0.00001
Guo 2022 [28]	<0.00001	89	Random	6.82	4.78 to 8.85	<0.00001
Li 2020 [32]	<0.00001	88	Random	7.00	5.11 to 8.88	<0.00001
Wang 2019 [36]	<0.00001	85	Random	7.02	5.27 to 8.76	<0.00001
Xian 2020 [33]	<0.00001	88	Random	6.54	4.63 to 8.45	<0.00001
Yang 2016 [40]	<0.00001	89	Random	6.47	4.65 to 8.28	<0.00001
Yuan 2019 [37]	<0.00001	89	Random	6.32	4.52 to 8.11	<0.00001
Zhang 2020 [34]	<0.00001	88	Random	7.02	5.18 to 8.86	<0.00001
Zhang 2021 [30]	<0.00001	87	Random	6.08	4.37 to 7.78	<0.00001
Zhao 2017 [39]	<0.00001	89	Random	6.42	4.64 to 8.21	<0.00001
Zhu 2018 [38]	<0.00001	89	Random	6.56	4.68 to 8.44	<0.00001
