# Supplementary material for: Clinical evaluation of platelet-rich plasma therapy for osteonecrosis of the femoral head: A systematic review and meta-analysis
Source: PLoS One. 2024 May 24;19(5):e0304096. doi: 10.1371/journal.pone.0304096 (PMC11125492; doi:10.1371/journal.pone.0304096)
Supplement: S4 Table — (PDF) [file pone.0304096.s004.pdf]

Supplementary table 4. Seneitivity analysis for visual analog scale score.

Eliminated study	Heterogeneity		Effect Model	MD	95% CI	P Value
	P Value	I^2 (%)				
None	<0.00001	85	Random	-0.75	-0.97 to -0.54	<0.00001
Chai 2022 [27]	<0.00001	86	Random	-0.74	-0.97 to -0.52	<0.00001
Dai 2019 [35]	<0.00001	86	Random	-0.77	-1.00 to -0.54	<0.00001
Guo 2022 [28]	<0.0001	75	Random	-0.69	-0.87 to -0.51	<0.00001
Li 2020 [32]	<0.00001	85	Random	-0.79	-1.01 to -0.57	<0.00001
Wang 2019 [36]	<0.00001	84	Random	-0.79	-1.01 to -0.58	<0.00001
Xian 2020 [33]	<0.00001	84	Random	-0.72	-0.94 to -0.49	<0.00001
Yang 2016 [40]	<0.00001	86	Random	-0.76	-0.98 to -0.54	<0.00001
Yang 2022 [29]	<0.00001	86	Random	-0.74	-0.97 to -0.51	<0.00001
Yuan 2019 [37]	<0.00001	86	Random	-0.76	-1.00 to -0.52	<0.00001
Zhang 2020 [34]	<0.00001	86	Random	-0.74	-0.97 to -0.51	<0.00001
Zhang 2021 [30]	<0.00001	86	Random	-0.74	-0.97 to -0.51	<0.00001
Zhu 2018 [38]	<0.00001	84	Random	-0.80	-1.01 to -0.58	<0.00001
