# Supplementary material for: Clinical evaluation of platelet-rich plasma therapy for osteonecrosis of the femoral head: A systematic review and meta-analysis
Source: PLoS One. 2024 May 24;19(5):e0304096. doi: 10.1371/journal.pone.0304096 (PMC11125492; doi:10.1371/journal.pone.0304096)
Supplement: S5 Table — (PDF) [file pone.0304096.s005.pdf]

Supplementary table 5. Publication bias evaluated by egger test.

Outcomes	Number of trails	Egger's test (P value)
Harris hip score	13 [27-28,30-40]	0.071
Visual analog scale score	12 [27-30,32-38,40]	0.368
