# Supplementary figures and images for: Clinical evaluation of platelet-rich plasma therapy for osteonecrosis of the femoral head: A systematic review and meta-analysis
Source: PLoS One. 2024 May 24;19(5):e0304096. doi: 10.1371/journal.pone.0304096 (PMC11125492; doi:10.1371/journal.pone.0304096)

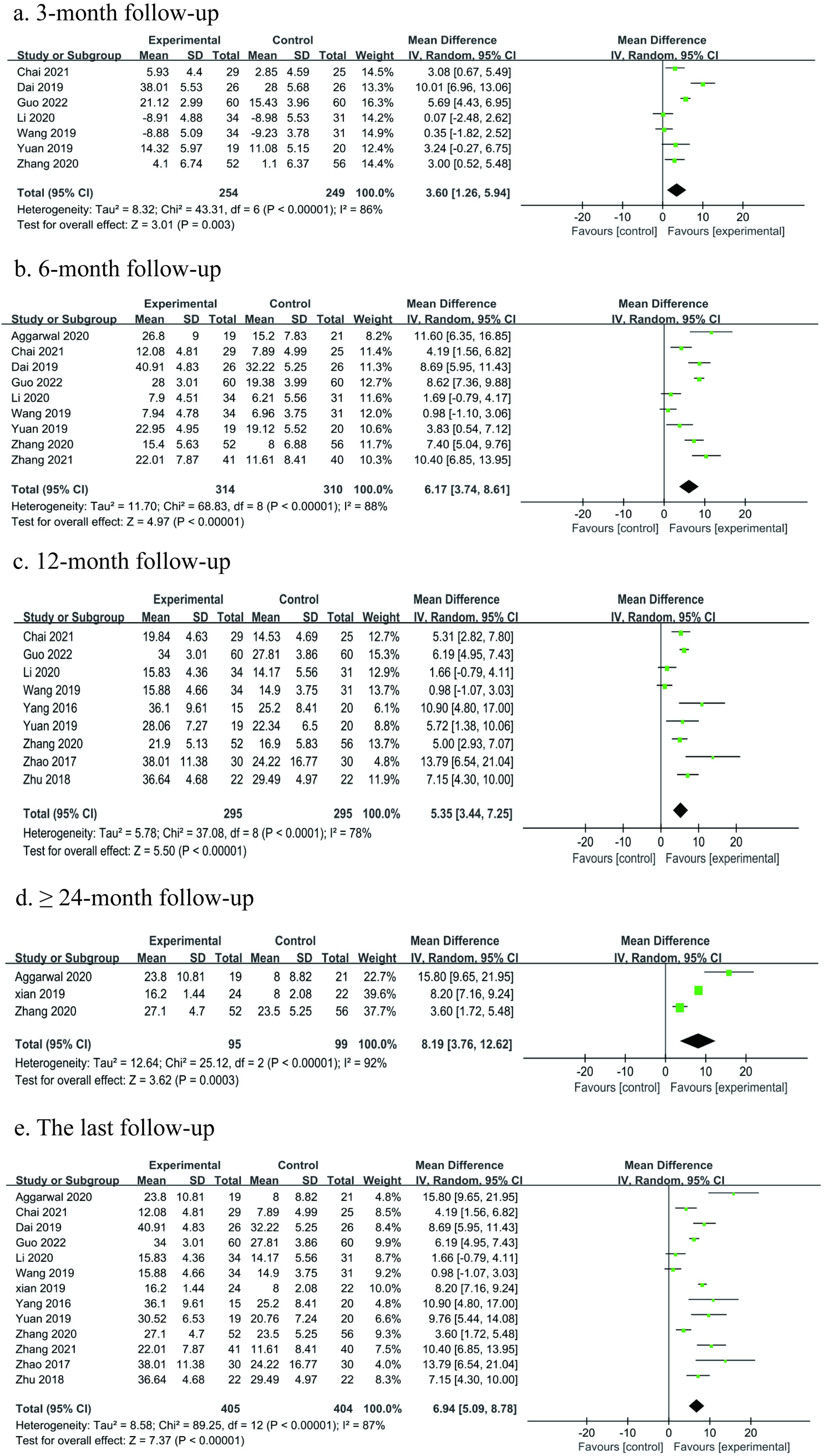

Supplement: S1 Fig — (TIF) [file pone.0304096.s006.tif]

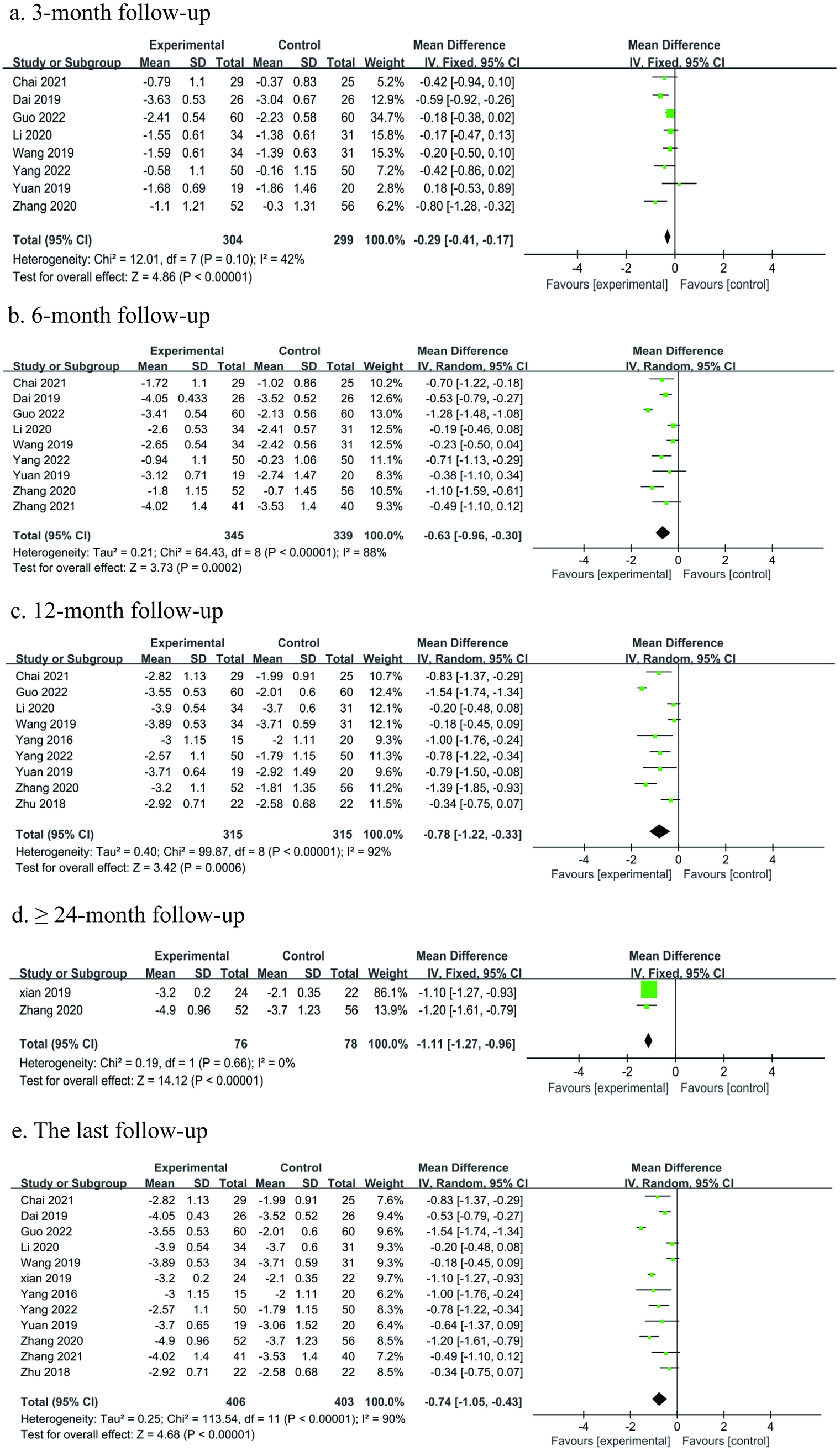

Supplement: S2 Fig — (TIF) [file pone.0304096.s007.tif]

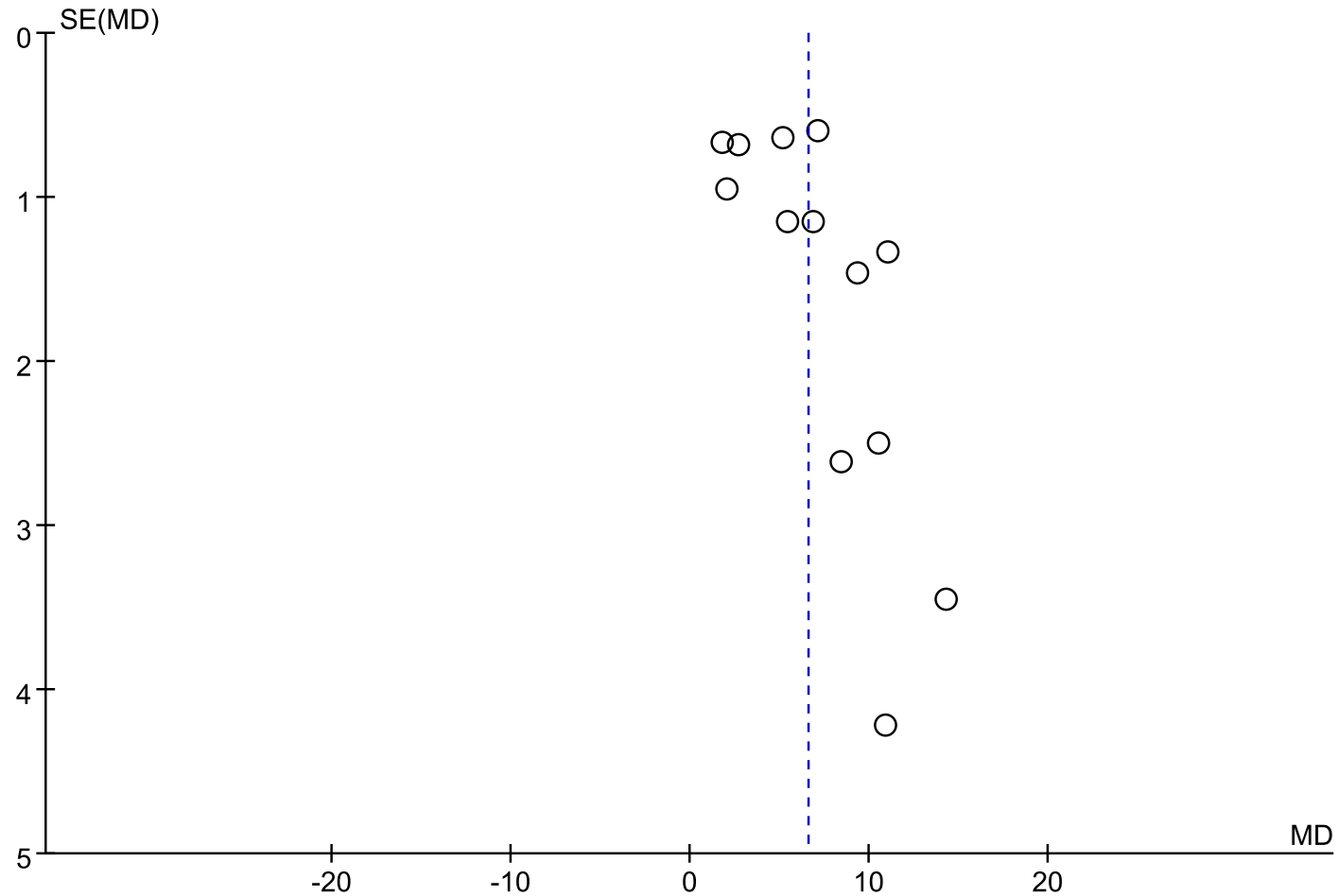

Supplement: S3 Fig — (TIF) [file pone.0304096.s008.tif]

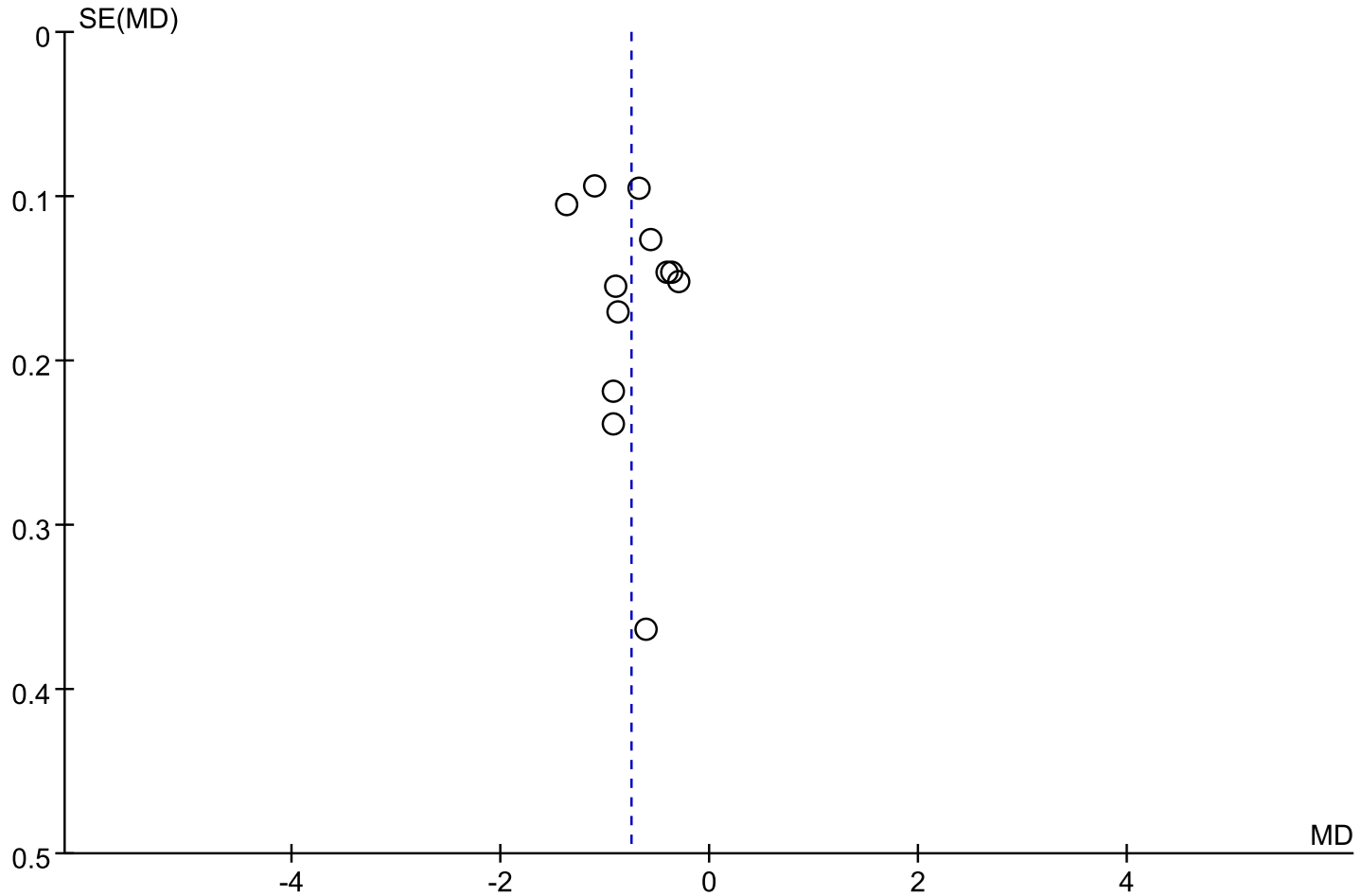

Supplement: S4 Fig — (TIF) [file pone.0304096.s009.tif]
